# Asymmetric Adaptive Fusion in a Two-Stream Network for RGB-D Human Detection

**DOI:** 10.3390/s21030916

**Published:** 2021-01-29

**Authors:** Wenli Zhang, Xiang Guo, Jiaqi Wang, Ning Wang, Kaizhen Chen

**Affiliations:** Faculty of Information Technology, Beijing University of Technology, Beijing 100124, China; guoxiang@emails.bjut.edu.cn (X.G.); jiaqiwang_jq@emails.bjut.edu.cn (J.W.); wangn@emails.bjut.edu.cn (N.W.); kzChen@emails.bjut.edu.cn (K.C.)

**Keywords:** RGB-D human detection, asymmetric, two-stream network, multimodal, adaptive fusion

## Abstract

In recent years, human detection in indoor scenes has been widely applied in smart buildings and smart security, but many related challenges can still be difficult to address, such as frequent occlusion, low illumination and multiple poses. This paper proposes an asymmetric adaptive fusion two-stream network (AAFTS-net) for RGB-D human detection. This network can fully extract person-specific depth features and RGB features while reducing the typical complexity of a two-stream network. A depth feature pyramid is constructed by combining contextual information, with the motivation of combining multiscale depth features to improve the adaptability for targets of different sizes. An adaptive channel weighting (ACW) module weights the RGB-D feature channels to achieve efficient feature selection and information complementation. This paper also introduces a novel RGB-D dataset for human detection called RGBD-human, on which we verify the performance of the proposed algorithm. The experimental results show that AAFTS-net outperforms existing state-of-the-art methods and can maintain stable performance under conditions of frequent occlusion, low illumination and multiple poses.

## 1. Introduction

With the rapid development of smart buildings and smart security, human detection in indoor environments has gradually become a popular research topic in the fields of computer vision and artificial intelligence. However, people can suffer from occlusion as well as variations in illumination, scale, and background, which make human detection in indoor scenes a challenging task. Methods based only on RGB features [1,2,3,4,5,6,7,8,9,10,11,12,13] can no longer meet the needs of human detection in many scenarios. With the popularization of inexpensive depth acquisition equipment, detecting human with the help of depth information has become an effective and feasible scheme. Unlike traditional RGB cameras, depth sensors in RGB-D cameras [14,15] do not rely on natural light sources and show strong robustness to changes in illumination. Depth images capture the distance from each people to the camera, thereby allowing the position relationships between objects to be calculated. Depth sensors thus provide a new strategy for addressing occlusion, lighting changes, and complex backgrounds. On this basis, the available human detection methods can be divided into three categories—methods based only on RGB data, methods based only on depth data and methods based on combined RGB-D data. The following sections describe and summarize related work in each of these three categories.

### 1.1. Based Only on RGB Data

Human detection can be regarded as a special case of object detection [1,2,3,4,5,6]. Depending on their structure of implementation, object detection algorithms based on RGB data can be divided into two categories: one-stage and two-stage methods. One-stage algorithms include YOLOv3 [1], RFB-Net2 [2], and CenterNet3 [3]. In these methods, each input image was divided by grid. In each grid cell, a series of anchors with different scales and ratios is established for extensive sampling of feature maps, which are finally used as the basis for bounding box regression and object classification. Representative algorithms with two stages include faster region-based convolutional neural network (Faster R-CNN) [4], Cascade R-CNN [5], and TridentNet [6]. In these algorithms, candidate region proposals are extracted in the first stage. Then, in the second stage, objects are regressed and classified based on these region proposals.

To address the challenges of occlusion and scale changes in RGB images, several pedestrian detection algorithms [7,8,9,10,11,12,13] have been developed based on novel processing approaches. Andre et al. [7] proposed a cascaded aggregate channel features (ACF) detector to accurately detect humans. Cheng et al. [8] divided the human body into multiple body parts and introduced a fast fused part-based model (FFPM) for pedestrian detection in a crowded environment. Tesema et al. [9] extended the Faster R-CNN framework to combine handcrafted features and CNN features for pedestrian detection. Wang et al. [10] introduced a RepLoss loss function to detect humans. Yang et al. [11] designed a part-aware region-of interest (RoI) pooling module to mine body parts with different responses. To handle varying levels of people occlusions, Xie et al. [12] used a graph convolutional network (GCN) to explicitly capture both inter- and intrapart co-occurrence information of different human body parts. Fawad et al. [13] first used background subtraction to obtain many human region proposals in a dynamic scene and then constructed aggregate channel feature vectors to describe these region proposals.

### 1.2. Based Only on Depth Data

With the emergence of low-cost depth sensors, human detection in depth images has become popular. Zhao et al. [16] extracted head region proposals from depth images and classified them by training a convolutional neural network (CNN). Zhang et al. [17] searched for extreme points on the contours of depth images to located head regions. As the second step, the head-shoulder descriptor (HSD) was constructed to jointly encode depth difference information and local geometric information. FUJIMOTO et al. [18] used the roundness and size of a height-continuous region to describe the human upper-back shape. They utilized implicitly included information in the missing region to alleviate the influences of partial loss of depth information caused by occlusions. Wetzel et al. [19] used a probabilistic framework to fuse the full multiview images from multiple sensors to handle occlusion and measurement noise in human detection. Tian et al. [20] jointly encoded a depth map, a multilayer depth template and a height difference map into three information channels, which were then sent to an AlexNet model for classification.

### 1.3. Based on Combined RGB-D Data

RGBD-based human detection methods generally utilize the depth information provided by depth images in combination with the color information provided by RGB images to perform multimodal feature fusion or joint decision making. Sun et al. [21] first extracted human heads from the projection depth image for human detection. Huang et al. [22] extracted histogram of orientation gradient (HOG) features and Haar-like features from color images and depth images, respectively. The method [23] consisted of a CNN and recurrent neural network (RNN) to learn distinctive and translationally invariant features from RGB-D data. Essmaeel et al. [24] used a multi-Kinect acquisition system to capture a complete target point cloud as a 3D human descriptor and classified people based on this descriptor. Lian et al. [25] proposed a regression guided detection network (RDNet) for RGB-D crowd counting, which uses the density map obtained by regression as the probability of head classification. The method in Reference [26] uses a new PEI representation to segment region proposals from RGB-D images and uses multistage filtering detection to reduce the number of candidate boxes. In References [27,28], the author searched for head-top points on depth images and extracted joint histogram features from RGB and depth images.

Some detection methods using RGB-D two-stream network have achieved a good result. Ophoff et al. [29] explored the best fusion position of RGB and depth information in the CNN, from which they concluded that the best results can be obtained by feature fusion towards the mid to late layers. Gupta et al. [30] proposed a depth map encoding method called HHA(horizontal disparity, height above ground, and angle with respect to gravity), which can encode the depth map into a three-channel image like RGB images. Zhang et al. [31] incorporated color information and 3D physical structure information to build a novel multichannel color shape descriptor (MCSD) to verify the candidate human regions. Zhang et al. [32] constructed a multi-stream network for extracting optical flow, depth and RGB features, and then connect feature channels from different modalities in fully connected layers. Zeng et al. [33] first constructed and trained RGB-CNN and depth-CNN networks, and then trained multimodal feature learning networks to fine-tune parameters.

The above methods have achieved promising results. However, manually designed RGB-D features are overly dependent on prior knowledge, and it is difficult to achieve end-to-end detection based on such features. The traditional two-stream CNNs used to process RGB-D data generally have a symmetric structure, meaning that the RGB branch has the same structure as the depth branch. However, with increasing model complexity, overfitting can easily occur, which can lead to partial loss of significant depth features. In addition, an inefficient RGB-D feature fusion strategy will not only lead to failure to fully utilize the auxiliary clues provided by depth images but also reduces the algorithm performance.

To overcome the disadvantages of the existing detection methods, this paper proposes a human detection method based on an asymmetric adaptive fusion RGB-D two-stream network called AAFTS-net. The main contributions of this paper are as follows:

(1) We propose an asymmetric two-stream network for RGB-D human detection, which reduces the complexity of typical symmetric two-stream networks while helping to overcome the partial loss of significant depth features in deeper layers and the insufficient representation of RGB features in shallow layers.
(2) We propose a multiscale fusion structure (Depth-FPN) for the depth stream, which can effectively fuse multilevel features from depth images.
(3) We introduce an adaptive channel weighting module (ACW module) for multimodal data fusion, which helps to select more valuable multimodal RGB-D feature channels for weighted fusion.

## 2. Method

**Figure 1:**
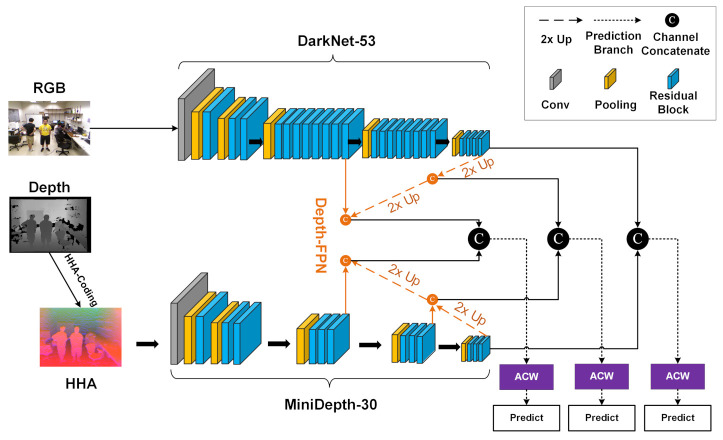
Overview the structure of the proposed asymmetric adaptive fusion two-stream network (AAFTS-net) for RGB-D human detection

This paper proposes an asymmetric adaptive fusion RGB-D two-stream network called AAFTS-net, with the aim of fusing features from RGB images and depth images. The proposed algorithm can efficiently and robustly detect indoor people under conditions of frequent occlusion, low illumination and multiple poses. The overall architecture of AAFTS-net is shown in Figure 1. The characteristics of this algorithm are as follows.

(1) Asymmetric two-stream network: The network structure consists of two parallel branches, an RGB branch and a depth branch. The RGB branch is based on Darknet-53 from YOLOv3, which consists of 53 convolutional layers for extracting features from RGB images. For the depth stream, a pruned version of DarkNet-53 is adopted based on depth features; this architecture, which retains 30 of the original convolutional layers, is called MiniDepth-30.
(2) Depth-FPN: Following MiniDepth-30, we also introduce a feature pyramid structure called Depth-FPN, which can effectively combine deep semantic features and shallow detail features from depth images by means of an upsampling operation. The multiscale depth features are enhanced and extracted so as to exhibit a one-to-one correspondence with the YOLOv3 feature hierarchy on prediction branches of three different resolutions.
(3) ACW: Inspired by the design of the Squeeze-and-Excitation (SE) block [34], an adaptive channel weighting module called the ACW module is proposed for multimodal data fusion. This module assigns weights obtained through adaptive network learning to each multimodal RGB-D channel to realize the efficient selection and fusion of multimodal features on the three prediction branches. Multimodal RGB-D feature maps of different resolutions (high, medium, and low) are input into the classification and regression layers of the corresponding prediction branches to generate the confidence values and coordinates of the predicted bounding boxes.

### 2.1. Asymmetric RGB-D Two-Stream Network

A traditional two-stream network usually has a symmetric structure, which means that the RGB stream and the depth stream have identical backbone structures. RGB images contain richer low-level details (colors, surface textures, etc.) as well as some discriminative high-level local features (facial features, body parts, etc.). Depth images highlight midlevel features such as edges, outlines, and shapes of objects. For symmetric two-stream networks, overfitting is prone to occur when the network is too deep [35]. This can result in partial loss of significant midlevel features from depth images. By contrast, when the network is too shallow, it is difficult to extract discriminative high-level features from RGB images.

**Figure 2:**
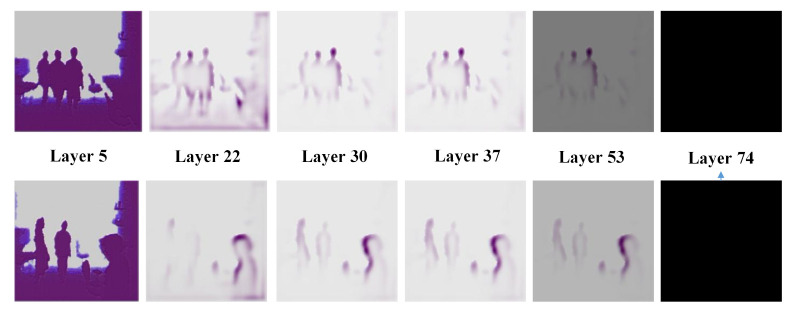
Convolutional feature visualization. The top and bottom rows depict the feature visualization results for the 5th, 22nd, 30th, 37th, 53rd, and 74th layers of the Darknet-53 network for two different depth image samples.

In this paper, we use a convolution visualization tool [36] to visually analyze the output feature maps from Darknet-53 to select a suitable network depth. We select the 5th, 22nd, 30th, 37th, 53rd, and 74th layer as output nodes to observe the object features learned from shallow, middle, and deep layers of DarkNet-53. The visualization results for two depth images are shown in Figure 2. Near the middle layers of Darknet-53 (for example, layers 30 and 37), more prominent edge contour features can be captured, while background and nontarget information can be effectively suppressed. In shallow layers (such as layers 5 and 22), the captured features contain more background redundancy due to the insufficient nonlinear mapping capabilities of the network. When the network depth reaches layer 53 or higher, the gradient values are very small, which makes the network parameters difficult to update. It will result in an inability to distinguish between foreground and background from depth image.

**Figure 3:**
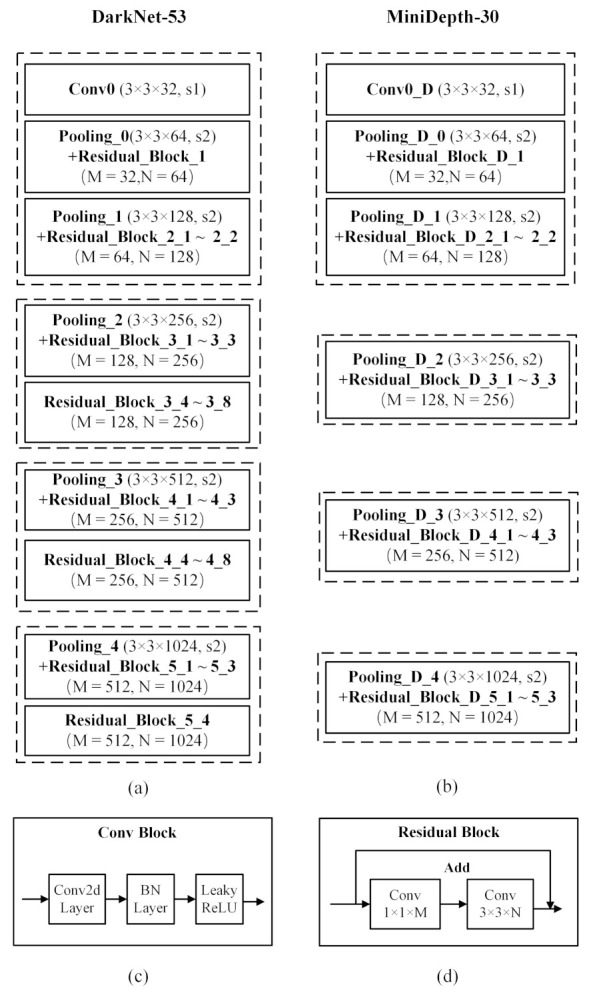
The structure of the asymmetric two-stream network proposed in this paper, showing of the network architecture and main components: (**a**) Darknet-53, (**b**) MiniDepth-30, (**c**) a convolutional layer, and (**d**) a residual block.

Based on this visual analysis, this paper proposes an asymmetric RGB-D two-stream network with the structure shown in Figure 3. The RGB stream is based on Darknet-53 and contains 52 convolutional layers. The structure of Darknet-53 is shown in Figure 3a. The depth stream is obtained by pruning Darknet-53 such that 30 convolutional layers are preserved; the resulting architecture is called MiniDepth-30. The network structure is shown in Figure 3b. The image input size for the DarkNet-53 and MiniDepth-30 networks are both 416 pixels wide and high.The general structure of a convolutional layer and a residual block are shown in Figure 3c,d. The numbers of residual blocks in the four sections of Darknet-53 are 3, 8, 8, and 4, respectively. For MiniDepth-30, we reduce the numbers of residual blocks in each of the last three sections to 3 and make the outputs dimensionally consistent with the feature maps output by the RGB stream. By reducing the model complexity in this way, the risk of overfitting can be reduced while increasing the speed of the inference process.

To select the optimal network depth for the depth stream, we also designed MiniDepth-22 and MiniDepth-40 using the same pruning method. We performed a control experiment using MiniDepth-22, MiniDepth-40 and Darknet-53 to verify the effectiveness of MiniDepth-30. The experimental results also prove the correctness of the proposed two-stream network, namely, that a network of moderate depth can generate higher-quality depth features.

### 2.2. Feature Pyramid Structure Following MiniDepth-30 (Depth-FPN)

Each pixel in a low-resolution feature map has a large receptive field and can be used to detect large-scale objects in the corresponding image. By contrast, a high-resolution feature map can retain more details of potential targets, which are beneficial for detecting small-scale objects. Therefore, the multiscale fusion of feature maps of different resolutions can greatly improve the robustness of detection for large, medium and small objects.

**Figure 4:**
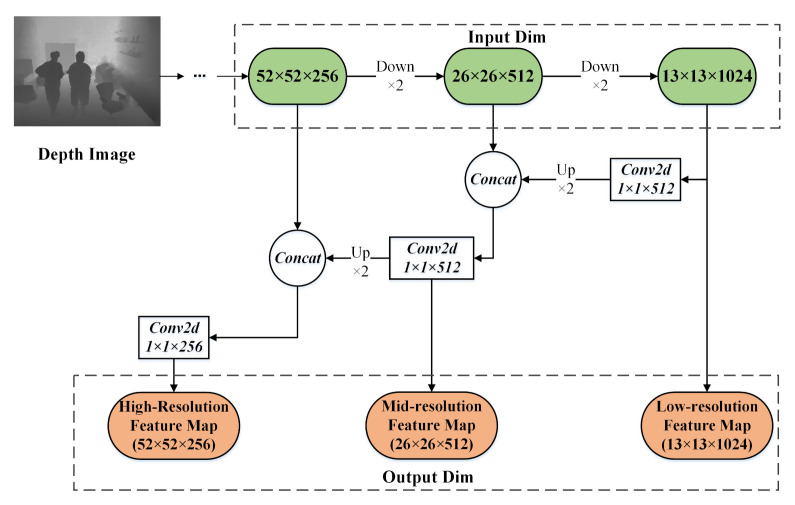
The structure of the Depth-FPN.

This paper proposes a depth feature pyramid structure called Depth-FPN, which follows MiniDepth-30 and combines high-order semantic features from low-resolution feature maps with low-order detail features from high-resolution feature maps. Depth-FPN is consistent with Darknet-53 in terms of the dimensions of the output feature maps. The structure of Depth-FPN is shown in Figure 4. For the 13 × 13 low-resolution feature map, a 1 × 1 convolutional layer is first used to eliminate the aliasing effect and reduce the number of output channels by a factor of 2. Then, the feature map size is magnified by a factor of 2 through an upsampling layer, and finally, the resulting feature map is concatenated with the channels from the 26 × 26 mid-resolution feature map. For the 26 × 26 feature map, the same operations described above are performed to obtain a high-resolution feature map with dimensions of 52 × 52. The feature maps at the three different scale (low, medium, and high) output by Depth-FPN have a one-to-one correspondence with the output of Darknet-53. The introduction of Depth-FPN overcomes the problem of the insufficient feature mapping capability for depth features at high resolution and the lack of detailed information at low resolution. This network design can promote the simple and efficient fusion of multimodal RGB-D data and improve the detection precision and recall for multiscale objects.

### 2.3. RGB-D Adaptive Channel Weighting (ACW)

**Figure 5:**
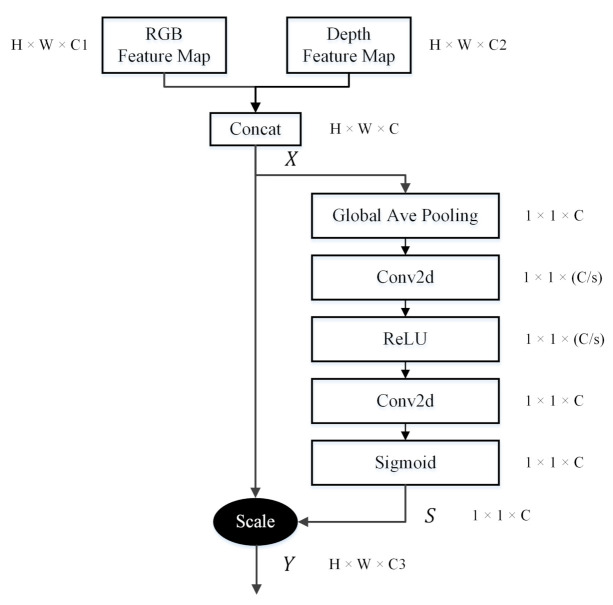
The structure of the Adaptive Channel Weighting (ACW) module.

The quality of the extracted multimodal RGB-D features and the feature fusion strategy usually determine the performance of RGB-D human detection. Since the RGB and depth features come from different data sources, they have different data distributions. Although a simple fusion method can be applied to explore the association between the RGB and depth data, such a method may also confuse the original ordering of the feature distributions. A large quantity of redundant information and a disordered feature distribution can seriously affect model convergence and detection performance. Thus, a more appropriate feature fusion strategy can enable more effective feature complementation and improve the quality of the multimodal features. Based on the idea of the SE block, this paper proposes a module for adaptive RGB-D channel weighting, named the ACW module, for RGB-D feature fusion. Focusing on the relationship among the various multimodal channels, we design a subnetwork to model the interconnections among the feature channels and adaptively learn to adjust their characteristic responses. Through this mechanism, the model can learn global information from the global receptive domain to achieve efficient feature selection. The architecture of the ACW module proposed in this paper is shown in Figure 5.

(1) The ACW module takes an RGB feature map and a depth feature map as inputs, both from the same pyramid level and with the same resolution. First, the two feature maps are concatenated in the channel dimension. The concatenated multimodal feature map is denoted by $X\in R^{H\times W\times C}$, where $C=C_{1}+C_{2}$.
(2) X is sent to the branch network to adaptively learn the weight values for each multimodal channel. Since each feature channel in X has been generated by a convolution kernel, the output response of each cell reflects only the local receptive field and does not consider context information outside of this range. Therefore, a global average pooling layer, $F_{p}$, is introduced in the branch network. The global spatial information of each feature channel is aggregated into a channel descriptor, as shown in Equation (1). In this equation, the statistic $Z\in R^{1\times 1\times C}$ represents the output response after aggregation, $X_{c}$ represents channel C of input feature map X, and $Z_{C}$ represents channel C of the output response.
  (1) $$Z_{c}=F_{p}\left(X\right)=\frac{1}{H\times W}\sum_{i=1}^{H}\sum_{j=1}^{W}X_{c}\left(i,j\right).$$
(3) To limit the complexity of the model, a bottleneck is added after $F_{p}$ to learn a nonlinear mapping. This bottleneck consists of a 1 × 1 convolution with a reduction ratio of 1/s, a ReLU activation, and a 1 × 1 convolution with an expansion ratio of s. Then, the sigmoid function is used to introduce a gating mechanism for learning the nonlinear interactions among channels, generating a total of C channel activation values between 0 and 1. Equation (2) describes the gating function $F_{S}$, where σ and δ represent the sigmoid and ReLU functions, respectively; h(x,w) describes the convolutional response before activation; $W_{1}\in R^{C\times (C/s)}$ and $W_{2}\in R^{(C/s)\times C}$ represent the parameters of the first and second convolutional layers, respectively; and the elements of $S\in [S_{1},S_{2},\ldots ,S_{C}]$ are the C channel weight values.
  (2) $$S=F_{S}\left(Z,W\right)=\sigma \left(h\left(Z,W\right)\right)=\sigma \left(W_{2}\cdot \delta \left(W_{1}Z\right)\right).$$
(4) The final output of the ACW module, $Y\in R^{H\times W\times C}$, is obtained as shown in Equation (3). First, the scalar values $S_{c}$ are multiplied by the feature map $X_{c}$, thus mapping the input feature map X to a unique set of channel weights S. The greater the channel weight value, the more prominent that channel’s contribution to the human detection process. Finally, the output feature map Y for each prediction branch are sent to the classification layer and coordinate regression layer of YOLOv3 for final detection.
  (3) $$Y_{c}=F_{Scale}\left(X_{c},S_{c}\right)=S_{c}X_{c}.$$

## 3. Experiment

### 3.1. Datasets

In this paper, we used six challenging public RGB-D datasets to verify the performance of our algorithm under different indoor environments. These RGB-D images were captured by Kinect in multiple indoor scenes, including stores, offices, restaurants, building corridors.

(1) CLOTH: This dataset was provided by Liu et al. [27]. It was acquired using a Kinect in a clothing store. The Kinect was placed 2.2 meters above the ground and at an angle of 30 degrees from the ground. For this study, 496 pairs of RGB-D images were selected from the original dataset.
(2) OFFICE: The OFFICE dataset was shot in an office environment and was provided by Choi et al. [35]. We selected 12 representative video sequences for use in this study, retaining 2209 pairs of RGB-D images.
(3) MOBILE: This dataset is also from Choi et al. [37]. It mainly collected images from scenes such as meeting rooms, corridors and restaurants. The challenges for human detection that are represented in this dataset include horizontal shooting angles, dynamic backgrounds, overlapping people, and changes in distance and lighting. For this study, we selected 8 video sequences of the MOBILE dataset where contains a total of 691 pairs of RGB-D images.
(4) DARK: This dataset was captured at night by Zhang et al. [17] and consists of 275 RGB-D images. In this scene, the people and backgrounds cannot be distinguished based on the low-light RGB images alone. We chose to use DARK to evaluate the human detection performance of our method in low-light or even dark conditions.
(5) MICC (Media Integration and Communication Center) people counting dataset(MICC): The MICC dataset provided by Bondi et al. [38] mainly consists of video surveillance images collected in crowded indoor conditions. This dataset contains three recorded video sequences: FLOW, QUEUE and GROUPS. It contains 1128 pairs of images. The entire MICC dataset consists of 3193 pairs of RGB-D images.
(6) EPFL (EPFL Laboratory) Pedestrian Dataset(EPFL): This dataset is provided by Bagautdinov et al. [39] and consists of two scenarios. EPFL-LAB was shot in a laboratory and contains a total of 250 pairs of images. EPFL-CORRIDOR was captured in the corridor of a school building and contains 8 video sequences comprising a total of 1582 pairs of RGB-D images. Severe occlusion and human scale changes are the greatest challenges presented by this dataset. For the current study, this dataset was selected to evaluate human detection performance under occlusion conditions.

### 3.2. Implementation Details

(1) Data preprocessing: We integrated the selected data from CLOTH, OFFICE, MOBILE, DARK and MICC into a large RGB-D human detection dataset called RGBD-human, which contains 6,864 pairs of RGB-D images. We randomly divided the data into training, evaluation and test sets at a ratio of 6:2:2, corresponding to 4118, 1373 and 1373 image pairs, respectively. In addition, we evaluated the performance of AAFTS-net under occlusion conditions on the EPFL dataset. We randomly divided the EPFL dataset into a training set and a test set at a ratio of 6:4, corresponding to 1099 and 733 image pairs, respectively. Data augmentation was applied to the training samples using color jitter, random translation and rotation, and horizontal flipping. The ground-truth coordinates were also correspondingly modified at the same time. The original depth images often contain many holes of missing data. The depth values at these holes are ’none’, resulting in large black patches in the depth images. In this study, the hole-filling method proposed by Zhang et al. [40] was used to repair the depth images. The restored depth images were preprocessed using HHA [30] encoding.
(2) Network details: In AAFTS-net, the RGB stream is initialized using the parameters of Darknet-53 pretrained on the ImageNet dataset. For MiniDepth-30 and the remaining layers, Gaussian random initialization is used. The scales and matching strategy for the anchors are consistent with those of YOLOv3. For human detection, we need to label ‘person’ objects; therefore, the number of categories in the classification layer is 1.
(3) Hyperparameters: AAFTS-net was implemented using PyTorch1.0 and trained for 20 hours on a system running Ubuntu 18.04 with an NVIDIA GTX 1080Ti GPU. The number of training epochs was set to 100, and the batch size was 8. The initial learning rate was 0.001, which was reduced to 0.0001 after 30 epochs and further reduced to 0.00001 after 80 epochs, with a momentum of 0.9 and a weight decay of 1 $\times \;10^{-3}$. The confidence threshold was set to 0.3, and the NMS threshold was set 0.4.

### 3.3. Evaluation and Analysis

#### 3.3.1. Performance Metrics

In this paper, we used the false positive per image–miss rate (FPPI-MR) curve as the main evaluation parameter and added precision, recall and the F1-measure as auxiliary evaluation parameters to evaluate the detection performance of our algorithm. The false positive per image (FPPI) represents the number of false positives (FP) per image, as shown in Equation (7), which was used to evaluate the false detection rate. N represented the total number of samples in the test set. The miss rate (MR) was defined as the ratio of the number of undetected objects to the total number of ground-truth objects, as shown in Equation (8), which was used to evaluate the miss rate. By setting different confidence thresholds, we obtained a set of FPPI-MR values so that we could draw FPPI-MR curves. The smaller the area under the FPPI-MR curve was, the better the performance of the detection algorithm.

The calculation formulas for precision, recall and the F1-measure are shown in Equations (4)–(6). The F1 measure is used to balance the precision and recall for human detection. A larger F1-measure value indicates that the algorithm achieves better detection performance.

(4) $$\mathrm{Precision}=\frac{\mathrm{TP}}{\mathrm{TP}+\mathrm{FP}}$$

(5) $$\mathrm{Recall}=\frac{\mathrm{TP}}{\mathrm{TP}+\mathrm{FN}}$$

(6) $$F1=\frac{2\times \mathrm{Precision}\times \mathrm{Recall}}{\mathrm{Precision}+\mathrm{Recall}}$$

(7) $$\mathrm{FPPI}=\frac{\mathrm{FP}}{\mathrm{FN}}$$

(8) $$\mathrm{MR}=\frac{\mathrm{FN}}{\mathrm{TP}+\mathrm{FN}}$$

#### 3.3.2. Comparisons with YOLOv3

To verify that the introduction of depth information in AAFTS-net is helpful for human detection, we compared the detection results achieved with AATFS-net and YOLOv3 on the RGBD-human, OFFICE, DARK and MICC. In the training phase, we used the same training strategy as RGBD-human to obtain the AAFTS-net and YOLOv3 models for experimental comparison. For YOLOv3, only RGB images were selected for training. During the test phase, we fixed the confidence threshold and NMS threshold to 0.3 and 0.4 to obtain the results shown in Table 1 and Table 2.

(1) Comparison results on RGBD-Human. The experimental results are compared in Table 1. The AAFTS-net model proposed in this paper achieved FPPI and MR values that were lower than those of YOLOv3 by 0.13 and 0.12, respectively, which shows that our algorithm can effectively reduce the false detection rate and missed detection rate. Additionally, the F1 measure increased by almost 2% and the precision increased by 3.7%, which confirmed that AAFTS-net is better than YOLOv3 at detecting accuracy. Compared to the original YOLOv3, we obtained the abovementioned significant performance improvement at the cost of only 0.003 ms inference time. The real-time speed of the AAFTS-net algorithm on NVIDIA 1080Ti reached 47 FPS. This improvement is closely related to the characteristics of the depth features. Depth images show strong anti-interference capabilities and robustness to occlusion. It also confirms with an appropriate feature construction mechanism, the information provided by depth features can be used to supplement and strengthen the information provided by RGB features, thus obtaining a more robust multimodal feature representation.
(2) Comparison results on OFFICE, DARK and MICC. In the RGBD-Human, each subset represented a different indoor detection scene. Different data distributions of each subset could affect algorithm performance. Therefore, we compared the results of AAFTS-net and YOLOv3 on OFFICE, DARK and MICC to analyze the influence of the metadata from different scenes and different levels of data quality on the detection results. The experimental results are shown in Table 2.

From the experimental results, we can see that the performance of AAFTS-net on the OFFICE, MICC and DARK datasets was better than that of YOLOv3. This strongly confirms that our proposed algorithm can be applied to human detection tasks in a variety of indoor scenarios with high accuracy. In MICC, the FPPI value of AAFTS-net decreased by 0.123, and the precision increased by 2% compared those of to YOLOv3, which indicates that our algorithm significantly reduces false positive errors during the detection process. Similar results also occured in OFFICE. It is worth noting that the performance improvement of our proposed algorithm on DARK was significant. FPPI and MR decreased by 0.383 and 0.237, respectively. At the same time, the F1 measure increased by more than 20%. This confirms that AAFTS-net can significantly enhance the detection robustness in dark conditions compared to traditional RGB-only methods.

#### 3.3.3. Comparisons with Other Existing Methods

**Figure 6:**
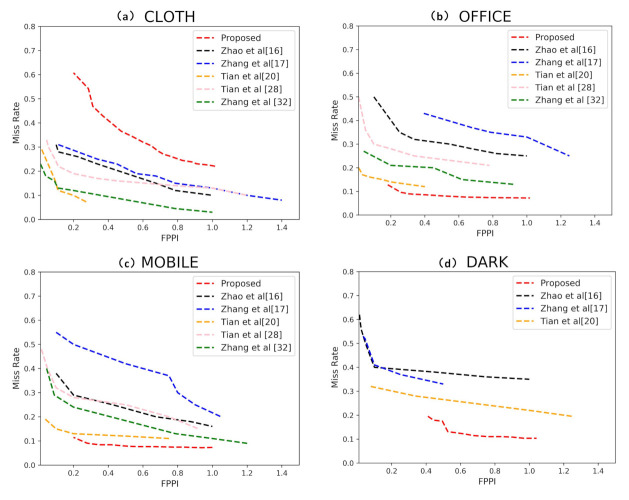
Comparisons of the performance of AAFTS-net and other state-of-the-art methods on four datasets: (**a**) CLOTH, (**b**) OFFICE, (**c**) MOBILE, and (**d**) DARK.

We compared the proposed AAFTS-net with the existing human detectors [16,17,20,28,32] on the CLOTH, OFFICE, MOBILE datasets and plotted the corresponding FPPI-MR curves respectively. To verify the performance of AATFS-net under low-illumination condition, we present the experimental results achieved on the DARK subset for comparison with the results [16,17,20]. The FPPI-MR curve on these four datasets are shown in Figure 6a–d. Since the source codes of comparison methods are not publicly available, we used the results reported by the authors of the respective articles.

The experimental results in Figure 6 show that our algorithm performed well on three public datasets except for CLOTH. The average MR values of our algorithm on OFFICE, MOBILE and DARK were all lower than 0.1, which illustrates that AAFTS-net can robustly detect humans in various restricted scenarios. At a fixed FPPI, our method had a lower average false detection rate and missed detection rate, which is significantly better than other existing methods [16,17,20,28,32]. Especially in the DARK dataset, the human eye can hardly see anyone. Compared with previous work [20], our algorithm improves the detection performance by nearly two times. The method [16] identifies head regions by considering the proportional relationship between depth values and real head size, but this coarse extraction method has a large positioning error, making it difficult to accurately locate the coordinates of human in images. The methods [17,20,28] search for the extreme points of head contours. However, these methods fail in cases of dense occlusion and complex backgrounds and rely heavily on the accuracy of the original depth data. The multistream network [32] has high model complexity, resulting in difficulty in reaching convergence. In addition, only bounding boxes of a single size and aspect ratio are considered, meaning that the detector has poor adaptability to objects of different sizes. Moreover, only late fusion is performed, increasing the risk of feature redundancy and the false positive rate.

**Figure 7:**
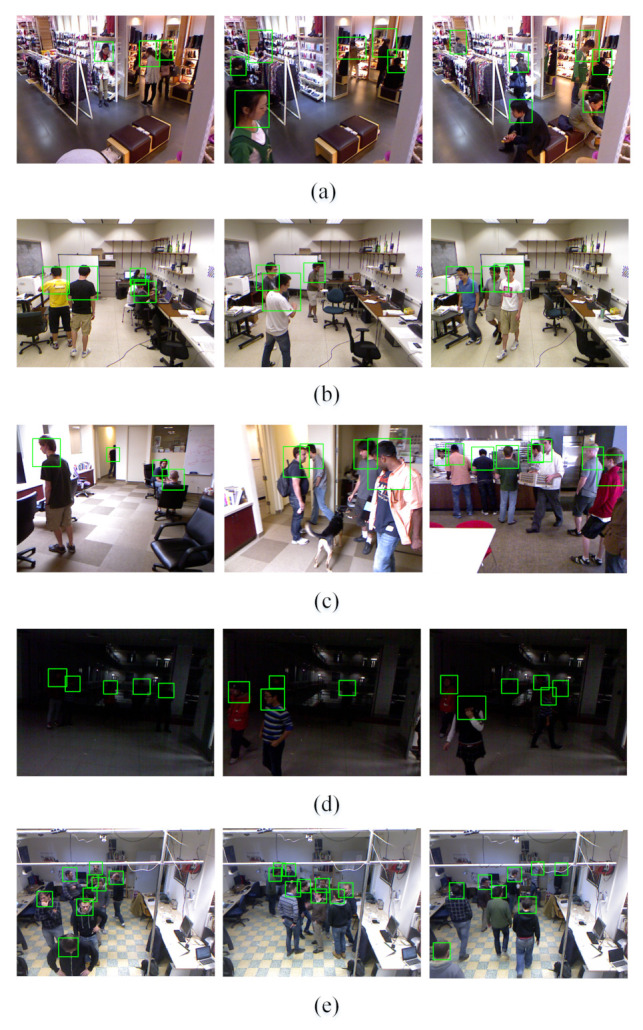
Results of AAFTS-net on each subset: (**a**) CLOTH, (**b**) OFFICE, (**c**) MOBILE, (**d**) DARK, and (**e**) MICC.

Compared with the above methods, we constructed a CNN-based detector through more refined feature fusion strategies, which improves the utilization efficiency of multimodal features. By introducing an asymmetric two-stream network, we can reasonably restrict the model complexity of the network to take full advantage of the depth features while ensuring a fast algorithm speed. The feature pyramid structure and channel weighting mechanism emphasize the commonalities and differences between data of different modes. These improvements can effectively handle challenging conditions such as partial occlusion, low illumination, and multiscale targets while maintaining high detection accuracy. Some more intuitive examples of detection results obtained by AAFTS-net is shown in Figure 7.

As shown in Figure 6a, the FPPI-MR results are slightly worse on the CLOTH dataset. According to our analysis, the main reason for this lower performance is that there is an excessive amount of depth information missing in the original CLOTH dataset, and these missing regions are mostly concentrated at the locations of human targets. Thus, the poor quality of the depth images makes it difficult to extract effective depth features for human detection.

#### 3.3.4. Occlusion Performance

Crossing occlusion is a universal challenge in human detection. Occluded people are only partially visible, which may lead to missed detections and incorrect detections. In this study, we verified the human detection performance of AAFTS-net under conditions of frequent occlusion on the EPFL dataset. EPFL includes various types of occlusion, such as long-distance occlusion, crossing occlusion, multiple poses, and severe occlusion. The dataset was divided into a training set and a test set at a ratio of 6:4, with the training set consisting of a total of 1070 pairs of RGB-D images for training the AAFTS-net and YOLOv3 human detectors. YOLOv3 was trained on only the RGB images as a control.

**Figure 8:**
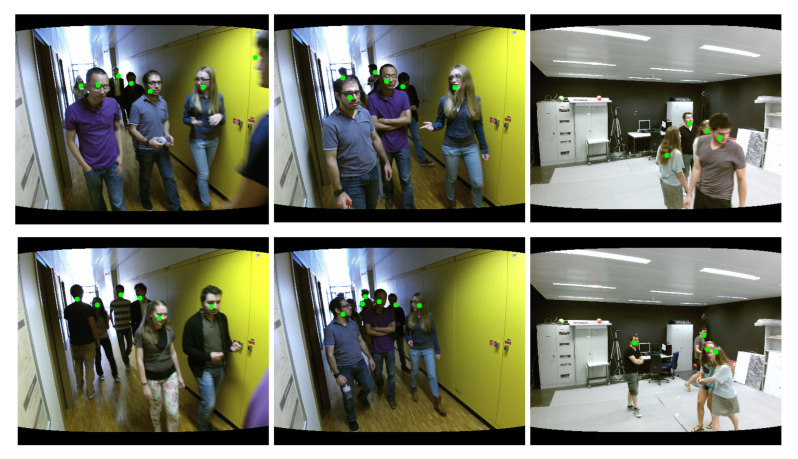
Detection results obtained under conditions of frequent occlusion, where the green points are the center points of the predicted bounding boxes.

The experimental results are shown in Table 3. AAFTS-net shows significantly higher robustness to human occlusion compared to YOLOv3. People at different distances from the camera are associated with different depth values in the depth images. Consequently, our approach can efficiently exploit this information to overcome the challenge presented by the inconspicuous individual depth differences in the RGB images and generate discriminatory features for distinguishing occluded individuals. Thus, the AAFTS-net algorithm achieves comprehensive improvement in performance, with 7% higher accuracy, a 2.5% higher recall rate, and an effectively reduction in the occurrence of false detections and missed detections under frequent occlusions. A visualization of the results for several samples from EPFL are shown in Figure 8. Due to the large amount of human occlusion, we mark only the center points of the predicted bounding boxes to clearly represent the detection results.

#### 3.3.5. Ablation Study

(1) Selection of the asymmetric network structure: We used the same pruning strategy used for MiniDepth-30 to construct MiniDepth-22 and MiniDepth-40, which retain 22 and 40, respectively, of the convolutional layers in Darknet-53. In this study, MiniDepth-22, MiniDepth-30, MiniDepth-40 and Darknet-53 were each used as the basis of the depth stream in the RGB-D two-stream network, and the corresponding networks were all trained and tested on the RGB-human dataset. The results obtained with these different network structures are compared in Table 4. It can be seen from this table that MiniDepth-30 yielded the best results, while the results of the symmetric Darknet-53-based network were the worst. This finding also confirms our previous hypothesis that depth images and RGB images contain unbalanced amounts of information that can aid in human detection. Compared with MiniDepth-22 and MiniDepth-40, the optimal network depth achieved in MiniDepth-30 overcomes the lack of shallow feature extraction ability while ensuring the complete validity of the depth features.
(2) Component contributions: The individual contributions of the various components of AAFTS-net were evaluated through additional ablation experiments. The investigated components mainly include Depth-FPN, the ACW module and the use of HHA depth coding. The corresponding experimental results are shown in Table 5. The introduction of Depth-FPN results in a 1.2% improvement in the F1 measure performance of AAFTS-net, thus demonstrating that multiscale feature fusion enhances the detector’s adaptability to human targets of different sizes. HHA coding provides a small improvement in the performance of the algorithm, with an increase of only 0.7%. Its main benefit is related to the enhancement of the edges of human targets in the depth images. The ACW module gives AAFTS-net a 3.5% performance boost, showing that our exploration of the intrinsic correlations between the RGB and depth feature channels leads to an obvious performance improvement. Our simple and efficient feature selection strategy can improve the fusion efficiency for multimodal data and generate a high-quality feature representation for RGB-D human detection.

## 4. Conclusions

In this paper, we propose an asymmetric adaptive fusion two-stream network named AAFTS-net, which is used to improve the performance of RGB-D human detection in complex scenes involving challenges such as frequent occlusion, low illumination and multiple poses. The proposed network design effectively reduces the model complexity of typical two-stream networks while improving the utilization of depth features. A depth feature pyramid structure (Depth-FPN) is proposed, which uses context information to enhance the multiscale representation of depth features. In addition, we introduce an adaptive channel weighting (ACW) module to determine the weights for the combination of the RGB-D feature channels to ensure efficient feature selection and information complementation. The experimental results obtained on the RGBD-human dataset and the EPFL occlusion dataset show that AAFTS-net achieves state-of-the-art performance and maintains high robustness under conditions of frequent occlusion, low illumination, and multiple poses. In the future, we will attempt to select lighter models and more efficient coding methods for depth images, with the aim of further improving the real-time speed and accuracy of the algorithm. Additionally, we will consider further optimizing our algorithm under outdoor sequences to improve its outdoor adaptability.

## Figures and Tables

**Table 1:** Comparison of the results of AAFTS-net and YOLOv3 on RGBD-Humans.

Methods	FPPI	MR	Precision	Recall	F1 Measure	Inference Time
YOLOv3 (Only RGB)	0.378	0.095	0.913	0.905	0.908	0.020 ms
AAFTS-net (Ours)	0.259	0.083	0.940	0.917	0.928	0.023

**Table 2:** Comparison of the results of AAFTS-net and YOLOv3 on OFFICE, DARK and MICC.

Dataset	Num	Method	FPPI	MR	Precision	Recall	F1 Measure
OFFICE	881	YOLOv3	0.363	0.124	0.878	0.876	0.877
		AAFTS-net	0.270	0.121	0.906	0.879	0.893
DARK	95	YOLOv3	1.210	0.406	0.686	0.594	0.637
		AAFTS-net	0.827	0.169	0.817	0.831	0.824
MICC	1301	YOLOv3	0.356	0.038	0.939	0.962	0.950
		AAFTS-net	0.233	0.037	0.959	0.963	0.961

**Table 3:** Comparison of the results of AAFTS-net and YOLOv3 on the EPFL occlusion test set.

Method	FPPI	MR	Precision	Recall	F1 Measure
YOLOv3 (Only RGB)	1.190	0.133	0.803	0.867	0.834
AAFTS-net (Ours)	0.664	0.108	0.870	0.892	0.880

**Table 4:** Evaluations for network depth selection.

	MiniDepth-22	MiniDepth-30 (Ours)	MiniDepth-40	Darknet-53
FPPI	0.284	0.259	0.301	0.285
Miss rate	0.099	0.084	0.093	0.097
Precision	0.924	0.941	0.919	0.915
Recall	0.901	0.916	0.907	0.903
F1 measure	0.914	0.928	0.912	0.909

**Table 5:** Evaluation of the contributions of each important component of AAFTS-net.

	HHA + ACW	PN + ACW	HHA + FPN	AAFTS-Net
Depth-FPN?		✓	✓	✓
ACW?	✓	✓		✓
HHA?	✓		✓	✓
F1 measure	0.916	0.920	0.893	0.928
